# Multi‐Organ Approaches to Cultivated Meat Biomanufacturing: Conceptual Applications of Ruminal Fermentation and Co‐Cultures

**DOI:** 10.1111/1750-3841.71057

**Published:** 2026-04-06

**Authors:** Morgan Rease, Wasitha Praveen de Wass Thilakarathna, Chandni Praveen, Suresh Pillai, Reza Ovissipour

**Affiliations:** ^1^ Department of Food Science and Technology Texas A&M University College Station Texas USA

## Abstract

Current academic research and industrial processes for cultivated meat production primarily utilize cultures of single cell types (monocultures) that do not capture the complexity of the in vivo environment of muscle and fatty tissues in the body. These systems are simplified for operational practicalities and cost reduction rather than to improve product quality. Irrespective of the manufacturing process, a food product must be of high quality to survive in competitive markets. Co‐culture of multiple cell types is a well‐established practice that enhances tissue qualities by allowing the cells to exchange cytokines and nutrients and to engage in cell‐cell signaling. These synergies promote tissue function and phenotype stability and alter cellular and tissue composition. Co‐culture can improve processes by reducing the need for exogenous media components such as growth factors. Expanding this system to include the products of rumen fermentation, such as B‐vitamins and short‐chain fatty acid (SCFA) has potential to further improve culture conditions and product qualities, particularly for cultivated meat of ruminant species (e.g., cattle, goats, sheep, and lambs). In this review, we cover the role of multiple cell types (notably fibroblasts and liver cells) as well as the role of rumen fermentation products in supporting the development and quality of the desired muscle and fatty tissues. We identify the opportunities and highlight the challenges associated with connecting these cultures as modules in a multi‐organ bioreactor series to further enable synergies and improve product qualities as part of a novel, bio‐inspired, multi‐organ production system.

## 1 Introduction

The global population is increasing and is expected to reach approximately 9–10 billion people by 2050 (United Nations 2024). Dietary consumption of animal proteins as a proportion of protein intake has also been increasing over time (Drewnowski and Hooker 2025). With this broad dietary shift toward consuming more animal protein and a growing population, the global demand for animal proteins is on the rise. Relative to 2020s levels, meat demand is expected to increase by approximately 50%by 2050, and dairy demand is expected to increase approximately 30% (FAO 2011; Turk 2016; United Nations 2023). Despite intense consumer demand and a long history of production, industrial production of animal proteins at the levels now demanded is well known to be resource‐intensive, a major contributor to climate change, and associated with many environmental issues (FAO 2023; Hayek and Garrett 2018; Peters et al. 2007; Tuomisto and Ryynänen 2024; Xu and Lan 2016). Cellular agriculture, the production of animal proteins using fermentation and cell culture technologies, is an emerging field with potential to help overcome some of these challenges if scaled manufacturing can be achieved (Stout et al. 2022; Tuomisto and Ryynänen 2024).

One of the most significant challenges in cultivated meat production is the development of low‐cost media, which constitutes a substantial portion of the total production cost (Humbird 2021; O'Neill et al. 2021; Quek et al. 2024). The high cost of media, driven primarily by animal sera and growth factors, makes scale‐up work prohibitively expensive (Amirvaresi and Ovissipour 2024; Amirvaresi et al. 2025; Specht 2020; Stout et al. 2022, Stout et al. 2023; Quek et al. 2024). Despite significant advancements over the last several years in lower‐cost serum‐free media development for cultivated meat production, the relationship between culture media composition or other bioprocess parameters and organoleptic properties has not been widely published on (Quek et al. 2024; Stout et al. 2022). The limited number of publications on cell culture organoleptics that do exist indicate that there are deltas to close to produce products truly identical to conventionally produced meat products (Lew et al. 2024; O'Neill et al. 2021; Piantino et al. 2025). Furthermore, these studies are narrow in scope relative to organoleptic studies on other food commodities due to the highly limited quantities of final products. While some consumers may make initial purchases on novelty and positive perceptions of a product, repeat purchase behaviors, which are needed for long‐term success, are driven primarily by price and organoleptic properties (Lange et al. 1998; Mueller and Szolnoki 2010; Tijssen et al. 2019; Tomiyama et al. 2020). Research is still needed on understanding and controlling the qualities of the end products in terms of nutrition, flavor, and functionality.

Cultivated meat currently relies on simplified in vitro conditions that lack the complexity of the holistic support system of a living animal. In vivo, developing muscle cells are supported by a complex system of other cell types and organs. Connective tissue cells produce extracellular matrix (ECM) and paracrine factors (Thummarati and Kino‐Oka 2020). The liver metabolizes nutrients and detoxifies waste (Polidoro et al. 2021). In ruminants (e.g., cattle, sheep, goats, and deer) the microbiome of the rumen transforms feed into essential, bioavailable nutrients (González‐Montaña et al. 2020; Holman et al. 2024). Growing muscle, fat, or other cells in isolation within a static medium neglects these supportive interactions in pursuit of lower complexity and operationally simpler processes. As a result, cultured tissues may have suboptimal growth, require frequent media changes to remove waste, lack the biochemical cues that drive maturation in vivo, and lack specific compounds from ruminal fermentation that enhance the flavor of final products (Rosso et al. 2004).

This review will discuss multi‐organ and multi‐tissue approaches to cultivated meat production, such as direct co‐culture of cells from multiple tissue types (e.g., muscle, fat, connective tissue, and liver), indirect co‐culture of cells with conditioned media or a series of bioreactors, and incorporation of postbiotics from ruminal fermentation into the culture media. We hypothesized that integration of some of these complexities from bodily systems can provide meaningful, positive alterations to the qualities of cultivated products relative to monoculture systems. Co‐culturing can stimulate extracellular matrix interactions and provide growth factors to improve myogenesis and adipogenesis (Ben‐Arye et al. 2020; Hicks et al. 2012; Holt et al. 2010; Morikura et al. 2024; Xu et al. 2022). Ruminal fermentation postbiotics can provide microbial metabolites such as vitamins and short‐chain fatty acids (SCFAs) to alter the flavor and nutritional profile of the final products (Dinh et al. 2021; Holman et al. 2024; Zhang et al. 2022). We align recent literature with our hypothesis and the challenges it entails.

## 2 Co‐Cultures

### 2.1 Co‐Culture Approaches

In animals, tissues are composed of diverse cell types embedded within a complex ECM rich in structural and functional macromolecules. Interactions between cells and ECM (cell‐ECM) and interactions between the cells themselves (cell‐cell) are essential for regulating cellular processes, including proliferation and differentiation. Tissue composition (e.g., profiles of fatty acids, amino acids, sugars, minerals) and the temporal aspects of these various cellular processes affect the development of organoleptic and nutritional qualities (Acevedo et al. 2010; Bischoff et al. 2018; Chail et al. 2016; Dinh et al. 2021; Klemenz et al. 2019). Conventional, in vitro, monoculture systems lack these interactions, often resulting in suboptimal cell behavior and limited success in replicating functional tissue constructs (Bale et al. 2015; Haubner et al. 2015; Polidoro et al. 2021). In contrast, co‐culture systems involving two or more cell types can more closely mimic native tissue environments by facilitating multicellular interactions, thereby influencing cellular phenotypes, differentiation processes, cellular metabolism, and nutritional attributes of the final product (David et al. 2023; Rosso et al. 2004). Co‐culture techniques have been applied extensively in regenerative medicine for developing novel functional tissues or maintaining stem cell potency during expansion. In these systems, supporting cells can produce beneficial cytokines, growth factors, and ECM (David et al. 2023). For example, co‐culture of bovine satellite cells with smooth muscle or epithelial cells has shown enhanced myogenic differentiation and ECM protein expression compared to those that were monocultured (Ben‐Arye et al. 2020).

The two main approaches to co‐culture are direct co‐culture and indirect co‐culture. In the direct co‐culture systems, different cell types are cultured together with physical contact, enabling interactions through cell‐cell interactions, cell–ECM adhesion, and direct paracrine signaling. Indirect co‐culture systems are based on cell interactions without physical contact, relying on paracrine signaling through soluble factors (David et al. 2023). Indirect approaches may be particularly beneficial to the cultivated meat industry, as soluble factors can be generated separately and applied to current monoculture processes. Techniques like media conditioning, wherein cells of one type are grown and release soluble factors in a media that can then be filtered and used in other cultures, may replicate some of the benefits of the complex bodily systems discussed prior. Rather than formulating a medium that provides every component in an ideal form, this approach could leverage co‐cultures to naturally enrich and balance a basal medium, similar to how blood can be conditioned as it circulates through various organs of the body. One could envision a synergistic system using conditioned media that helps reduce the cost of serum‐free media by producing some of the more expensive components or boosting process efficiencies. However, the cost and practicality of running multiple cultures may negate those savings without optimization. In practice, this could take the form of an organ‐on‐a‐chip system (a microfluidic device for modeling interactions between organs in vitro) at a small scale or a series of bioreactors at larger scales, introducing hormonal and paracrine cues to otherwise isolated cell cultures, replicating some aspects of metabolism and inter‐organ signaling (Mandenius 2018; Ronaldson‐Bouchard et al. 2022). Media perfused through a liver module could be detoxified and enriched with hepatocyte‐derived molecules (e.g., albumin, IGF‐1), while fibroblast cultures could secrete adhesion proteins and cytokines into the medium, obviating the need for exogenous matrix coatings or serum. Co‐culturing may also provide cells with necessary growth factors or vitamins, reducing or eliminating the need for costly, purified components (Morikura et al. 2024; O'Neill et al. 2021; Piantino et al. 2025). Recent studies lend credence to this concept. Morikura et al. (2024) indicated that conditioned media from a direct co‐culture of human HepG2 liver cells and mouse NIH‐3T3 fibroblasts could be applied to bovine myogenic cells to enhance cell adhesion and proliferation in a serum‐free medium in uncoated culture dishes. Metabolite analysis showed that the co‐culture process enriched the medium with compounds like pyridoxamine (a form of vitamin B6), asparagine, and glutamic acid—nutrients that likely played a key role in supporting cell growth (Morikura et al. 2024).

### 2.2 Cell Types in Co‐Culture Systems

#### 2.2.1 Fibroblasts for Structure and Growth Support

**FIGURE 1:**
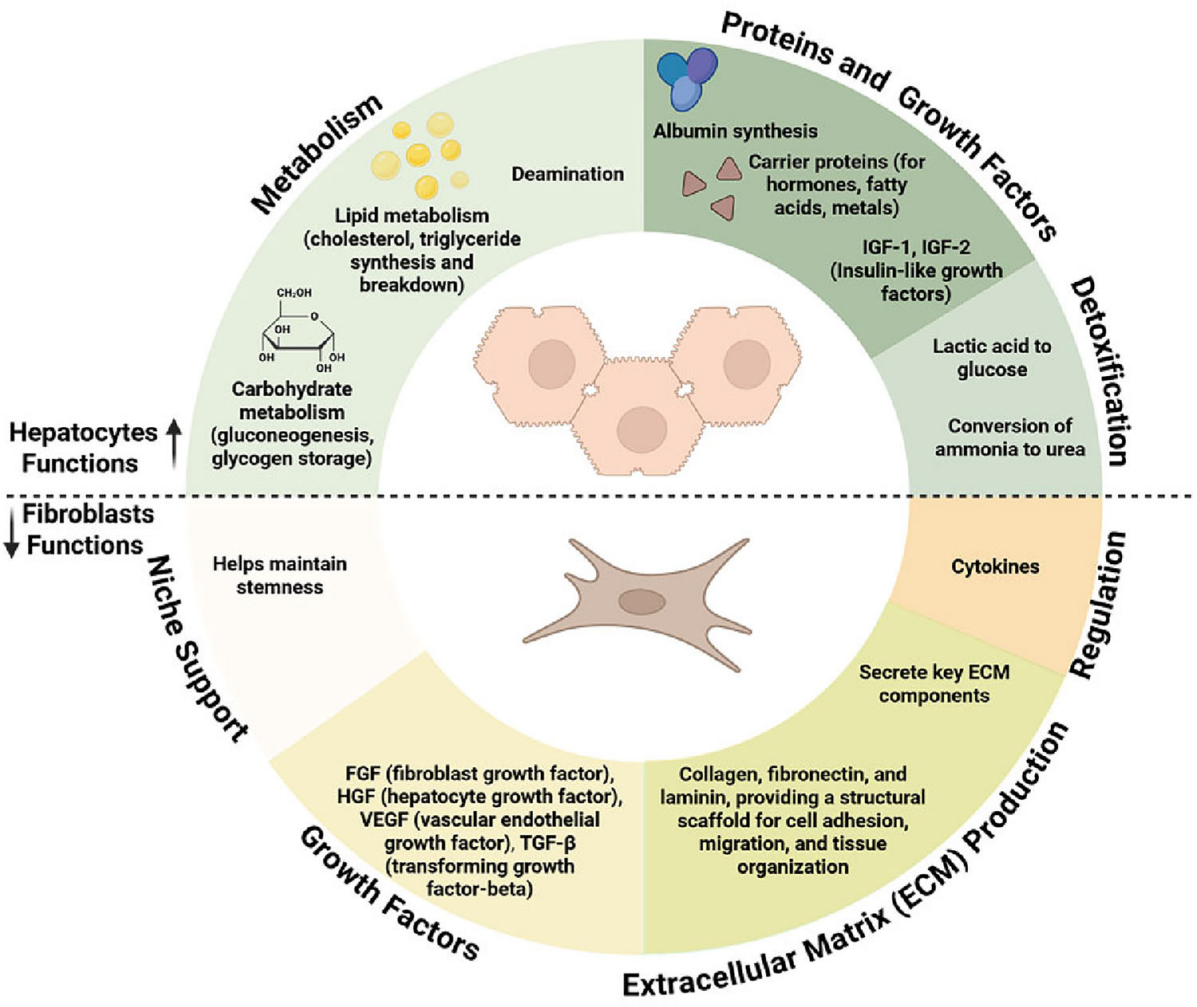
Overview of the cellular functions of fibroblasts and hepatocytes.

Fibroblasts are the principal cells of connective tissues and play a pivotal role in skeletal muscle development and regeneration. The functions and impact of fibroblast cells in tissues and cellular metabolism are presented in Figure 1.

In vivo, skeletal muscle fibers are surrounded by a framework of endomysium and perimysium connective tissue that is largely produced by fibroblasts, including specialized, muscle‐resident fibroblasts, which are often termed fibro‐adipogenic progenitors (FAPs) (Molina et al. 2021; Thummarati and Kino‐Oka 2020). These cells secrete cytokines and growth factors such as vascular endothelial growth factor (VEGF), hepatocyte growth factor (HGF), fibroblast growth factor (FGF), and transforming growth factor beta (TGF‐β). Also, they produce structural ECM proteins like type 1 and type 3 collagens, fibronectin, and proteoglycans. Collectively, these substances support the organized structure of muscle fibers and likely contribute to the texture of meat (Bomkamp et al. 2021; Duarte et al. 2013; Molina et al. 2021; Oppen et al. 2024; Roy and Bruce 2024; Thummarati and Kino‐Oka 2020; Xie et al. 2020). Achieving textural parity with conventionally produced meats will likely require cultivated meat to replicate this matrix composition and matrix architecture. Soluble factors, including various cytokines and growth factors, can be produced or enhanced by fibroblast cells to the benefit of other desirable cell types. HGF and VEGF, both of which can be secreted by fibroblasts, promote growth and vascularization in muscle and endothelial cells (Thummarati and Kino‐Oka 2020). HGF is well known as an activator of muscle satellite cells (muscle stem cells), stimulating their proliferation during muscle regeneration. VEGF, on the other hand, aids in angiogenesis. Co‐cultures of muscle cells with fibroblasts have shown increased VEGF secretion, which can enhance the formation of vascular networks (Thummarati and Kino‐Oka 2020). TGF‐β is also produced by fibroblasts. Alterations to protein expressions in myoblasts, such as the expression of collagen 1 or α‐smooth muscle actin (α‐SMA), can be observed when the two cell types are co‐cultured as well as when TGF‐β is supplemented directly (Krieger et al. 2018). Indirect co‐culture of fibroblasts with myoblasts has been shown to stimulate myogenic differentiation through interleukin‐6 (IL‐6). Improvements to myotube density and fusion index of C2C12 myoblasts indirectly co‐cultured with fibroblasts could be further enhanced via mechanical stretching of the fibroblasts relative to co‐cultures subjected to no strain (Hicks et al. 2012). Fibroblast growth factor 2 (FGF2), one of the many forms of FGF, is produced by fibroblasts and can enhance cell proliferation and muscle repair (Xie et al. 2020). Fibroblast‐derived ECM and soluble factors create a more nurturing niche for myogenesis than muscle cell monocultures would experience alone.

Fibroblasts produce ECM components such as structural proteins (e.g., fibrous collagen, and elastin), adhesive proteins (e.g., laminin and fibronectin), and ground substance components (i.e., glycosaminoglycans and glycoproteins) (David et al. 2023). Co‐culturing fibroblasts or fibroblast‐like cells with muscle cells has been found to improve outcomes in several studies (Hicks et al. 2012; Krieger et al. 2018; Thummarati and Kino‐Oka 2020). Fibroblasts can modulate the differentiation of myoblasts (muscle precursors) through both direct contact and secreted factors. Interestingly, the effects of fibroblasts on myogenesis can be bi‐directional. Low proportions of fibroblasts in co‐culture may enhance myoblast differentiation and alignment, whereas an excess of fibroblasts can lead to fibrosis or misalignment (Thummarati and Kino‐Oka 2020). Optimal ratios on the order of 30 percent fibroblasts have been reported to maximize pro‐myogenic factors without impeding muscle fiber formation (Thummarati and Kino‐Oka 2020). One common technique for taking advantage of the benefits of fibroblasts is to use them as a feeder layer. In this manner, the culture is distinguished from a direct co‐culture in that the fibroblasts have typically been rendered unable to proliferate. Fibroblasts are grown as an underlying layer to condition the growth surface and the culture medium. Use of fibroblast feeder layers of varying densities has been demonstrated to support hepatocytes, allowing for more prolonged growth and secretion of albumin relative to pure hepatocyte culture (Sakai et al. 2018).

Fibroblasts do not exclusively impact other cells; they themselves are affected by the culture conditions. Co‐culture of fibroblasts with adipose‐derived stem cells was found to improve the recovery of cultures exposed to varying levels of irradiation (Haubner et al. 2015). Fibroblasts were found to increase their expression of varying cytokines when exposed to macrophage‐conditioned media (Holt et al. 2010). The extent to which intramuscular preadipocytes, a type of fibroblast, were able to differentiate into adipocytes and accumulate lipids was decreased when grown in co‐culture with skeletal muscle satellite cells (Xu et al. 2022). Fibroblasts have also been transdifferentiated into muscle cells and made to accumulate lipids to replicate fatty tissues to achieve the varied benefits of multiple tissue types without co‐culture (Ma et al. 2024).

#### 2.2.2 Liver Cells for Growth Factors, Nutrient Conversion, and Detoxification

The liver is a metabolic powerhouse, performing hundreds of biochemical transformations that maintain homeostasis in animals (Figure 1). In a cultivated meat system, incorporation of liver cells into the overall production scheme could enable several key functions: synthesis of key compounds such as growth factors, hormones, and proteins; conversion of some nutrients into other more utilizable forms; and detoxification of inhibitory compounds and waste products (Polidoro et al. 2021). Primary liver cells are also notoriously difficult to culture for prolonged periods of time and quickly lose hepatic function, which could negate their theoretical beneficial contributions in a cultivated meat production system.

Serum albumin, or recombinant versions thereof, is a common additive to serum‐free culture media. In cost calculations for Beefy‐9 media, Stout et al. (2022) found recombinant albumin to be the first or second most cost‐contributing media component (depending on the level of added FGF) (Stout et al. 2022). As previously established, albumin is secreted by liver cells. Intuitively, if one could reduce or obviate the need for purified albumin to be added by producing it within the culture system that would be beneficial. We hypothesize that albumin synthesis from the inclusion of liver cells in a multi‐organ cultivated meat system is not a feasible approach given the current state of relevant technologies, though we discuss it briefly here for completeness. Stout et al. (2022) found 6.4 mg/mL of albumin to be an optimal balance of performance and cost, with increased albumin concentrations (as high as 11.2 mg/mL) resulting in better culture performance. Multiple studies have shown that albumin secretion varies over the duration of hepatocyte cultures (Bale et al. 2015; Miranda et al. 2010). Under optimal conditions and with stable production, the number of cells required to produce adequate albumin for 1 L of Beefy‐9 media with a 6.4 mg/mL albumin concentration would be approximately 2 × 10^11^. At such high quantities, adherent culture in flasks or as a subsystem in a culture is not feasible. One would likely use excessive quantities of media in pursuit of generating albumin for media supplementation from liver cells. Therefore, for hepatocyte culture to positively impact a multi‐organ cultivated meat production system specifically by producing albumin, significant development would be needed, and high‐density culture systems like perfusion or suspension cultures would need to be utilized.

Liver cells are known to produce many growth factors and, in some cases, are the primary producers of those growth factors in the body. Additionally, the growth factors in cell culture media are present at quantities orders of magnitude lower than proteins like albumin. Fetal bovine serum (FBS) contains >100 different proteins, among them are IGF proteins at levels of 500–900 ng/mL. Interestingly, Yamanaka et al. (2023) found that it is possible to obtain levels of IGF‐1 and IGF‐2 similar to that of standard 10% FBS media in media conditioned by RL34 rat liver epithelial cells. Moreover, the conditioned media were able to support the growth of bovine myoblasts (Yamanaka et al. 2023). Similar results have been reported by Chu et al. (2024) for C2C12 mouse skeletal muscle myoblast cells cultured in a medium conditioned by RL34 cells co‐cultured with a cyanobacterium. Co‐culturing with a cyanobacterium can significantly improve conditioned media for enhanced growth support by effectively removing waste products like lactate and ammonia through conversion of lactate to pyruvate and ammonia to amino acids (Chu et al. 2024).

The liver is central to nutrient metabolism and can convert various sugars, fats, and proteins from one form to another. For instance, alanine and glutamate can be interconverted to pyruvate and α‐ketoglutarate through alanine transaminase. In ruminants, the SCFA propionate can be converted to glucose through gluconeogenesis. The liver is also capable of de novo lipogenesis (DNL), though there are large differences between species on the extent to which DNL occurs. While the liver is the primary site of DNL in animals like humans and poultry, the liver is less essential for this purpose in pigs and ruminants due to DNL occurring in adipose tissues as well (Nguyen et al. 2008). Cholesterol, a vital lipid for cultivated meat production, can be generated via DNL and is among the many components in animal sera. Commercial serum replacements, such as Knockout Serum Replacement and AlbuMax, contain cholesterol, as it is often necessary for cell membrane health (Takii et al. 2022).

A key function of the liver is detoxification and neutralization of toxins, drugs, and deleterious metabolites. Many of these functions are catalyzed by specialized enzymes in the cytochrome P450 (CYP450) family produced by hepatocytes. CYP450 enzymes broadly catalyze phase 1 oxidation of many compounds (Polidoro et al. 2021). The Cori cycle and urea cycle in hepatocytes are critical for neutralizing cellular metabolic wastes through their transformation of highly toxic lactate and ammonia to glucose and the far less toxic urea, respectively. Cell culture media typically use pharmaceutical‐grade components, such as purified amino acids, to reduce impurities. However, high‐purity components contribute to the high cost of standard culture media formulations, and the use of lower‐purity food‐grade components is widely acknowledged to be necessary to achieve economically viable commercial production of cultivated meat. While the quantity and type of impurities vary, common impurities in pharmaceutical‐grade amino acids include elemental impurities, heavy metals, chlorides, ammonium, sulfates, and other amino acids (Karakawa et al. 2022). Food grade sources of amino acids or even broader protein hydrolysates can have similar types of impurities as well as residual compounds from the biological source materials and extraction processes. Even when impurities themselves are not toxic to cells, they can alter the cellular behavior in ways that may affect product quality or aspects of downstream processing (Ho et al. 2016). While elemental impurities and heavy metals would still pose hazards to the health and productivity of the culture, the incorporation of liver cells into a culture system could enable detoxification of some of these substances (e.g., biological source contaminants, ammonium) as well as toxic metabolites of other cells (e.g., ammonia, lactate) and thereby enable the critical step of using lower purity components.

#### 2.2.3 Ruminal Fermentation Products for Improved Nutrition and Organoleptic Qualities

Ruminants such as cattle, buffalo, sheep, goats, and lambs are important agricultural animals distinguished by the large digestive organ—the rumen. The rumen contains a diverse community of microbes, including bacteria, protozoa, fungi, and archaea, that work symbiotically to break down fibrous plant materials, transforming cellulose, hemicellulose, complex carbohydrates, and proteins into energy and nutrients needed by the animal (Langda et al. 2020). Urea produced by the liver when detoxifying ammonia can also be recirculated and passed into the rumen. Ruminal bacteria can break down urea back into ammonia to be utilized as a nitrogen source, even though mammals themselves cannot utilize urea and it is typically a waste excreted from the body in urine (Getahun et al. 2019). Ruminal fermentation also produces abundant gases, volatile fatty acids, B‐vitamins, mono and disaccharides, proteins, and biopeptides. The exact composition of the products of ruminal fermentation is considerably influenced by animal species, diet, and environmental conditions (Morgavi et al. 2013). In ruminant animals, ruminal fermentation has a significant impact on the vitamin content, the types and saturation of fatty acids, and the flavor of the products derived from those animals (Annison and Bryden 1998; Franco‐Lopez et al. 2020; Zhang et al. 2022).

Rumen microbes produce several B‐vitamins from the breakdown of the animal's diet. Among the B‐vitamins produced is vitamin B12, which is a crucial co‐factor for cell growth in addition to being an important vitamin for human nutrition. In fact, neither animals nor plants can synthesize vitamin B12, as it is exclusively synthesized by microbes. Ruminants rely on their rumen flora to produce vitamin B12 from dietary cobalt, which is then absorbed and used in metabolic pathways (e.g., odd‐chain fatty acid metabolism and methylation reactions) (González‐Montaña et al. 2020). Other B‐vitamins can be present in animal diets but are also synthesized to some extent by the ruminal community, highlighting the interplay of substrate and community in determining rumen fluid composition and animal nutrition (Seck et al. 2017). Many of these B‐vitamins are important enzyme cofactors or affect cellular processes. Riboflavin (B2) is a critical enzyme cofactor for many enzymes and is associated with the metabolism of carbohydrates, amino acids, and lipids. Nicotinamide (B3) functions as a kinase inhibitor and is a precursor to nicotinamide adenine dinucleotide (NAD^+^) (Schnellbaecher et al. 2019). Vitamin B3 forms are also associated with changes in ruminal community populations and intramuscular fat and marbling (Zeng et al. 2024). Vitamin B6, of which there are six forms with similar biological functions with pyridoxine HCl being the most common, enables coenzyme Q synthesis, gluconeogenesis, and various amino acid transformations (e.g., racemization, transamination, and elimination). Folic acid (B9) is a cofactor for purine and pyrimidine synthesis and amino acid metabolism (Meng et al. 2018; Schnellbaecher et al. 2019). While other B vitamins may not be meaningfully synthesized in the rumen, they can still be present in the rumen fluid as a result of the diet or substrate (e.g., the vitamin content of the feed or forage) for the ruminal community.

Diet and ruminal microbial consortia composition affect the ultimate fatty acids profile of ruminant meat. The diet of the animal contributes fatty acid to be incorporated into tissues, particularly long‐chain fatty acids, which are not produced by the ruminal community, and affects the population of ruminal communities as they respond to the abundance of different types of nutrients and conditions (Watkins et al. 2021; Zeng et al. 2024). Unsaturated fatty acids from the diet are hydrogenated in the rumen, a process referred to as biohydrogenation, increasing the abundance of saturated fats in the animal. Ruminal fermentation also produces many short‐chain and medium‐chain fatty acids (Dinh et al. 2021; Watkins et al. 2021; Zeng et al. 2024).

In ruminants, SCFAs such as acetate, propionate, and butyrate are highly impactful on animal nutrition, as SCFAs are a large source of metabolizable energy. In cattle, the SCFAs from rumen fermentation represent as much as 70% of metabolizable energy (Holman et al. 2024). Propionate in particular is an important substrate for gluconeogenesis (Kristensen et al. 1998). When propionate is in excess of the capacity of gluconeogenesis, it can be metabolized to branched and odd‐chain fatty acids, primarily by the liver and adipose tissue, respectively (Vlaeminck et al. 2006; Watkins et al. 2021). Acetate can be used directly as a substrate for lipogenesis (Holman et al. 2024). At low doses, butyrate can affect gene expression, inhibit proliferation, and promote differentiation of muscle cells (Wang et al. 2025).

The rumen's microbial consortia are variable, with bacteria being the most abundant, most diverse, and most studied. Ciliated protozoa and fungi are the next most abundant, with lesser amounts of archaea and others (Sanjorjo et al. 2023). Across multiple ruminant species, the bacterial phyla Bacteroidota and Firmicutes are typically highly abundant, as are the Prevotella and Bacteroides genera (Langda et al. 2020; Sanjorjo et al. 2023; Zeng et al. 2024). Neocallimastigomycota, Ascomycota, and Basidiomycota are common fungal phyla across ruminants, with Neocallimastigomycota and Ascomycota as the most abundant in sheep and goats (Langda et al. 2020; Sanjorjo et al. 2023). Ciliated protozoa make up a substantial portion of the estimated rumen microbial biomass (up to 50%) but seem to be less diverse relative to bacteria. Across multiple species, Entodinium is the most common protozoan genus (Sanjorjo et al. 2023). Additionally, despite their substantial presence and a long history of study (rumen protozoa were first described in 1843), their exact contribution to rumen function is unclear. Protozoa can break down plant materials, and they contribute significantly to methanogenesis (Newbold et al. 2015). Removal of protozoa from the community, a process known in this context as defaunation, can affect the animal in multiple ways, such as increasing the abundance of microbial protein by 30% and decreasing the abundance of other fibrolytic organisms (e.g., fungi, Ruminococcus spp.). (Newbold et al. 2015). The roles of fungi and bacteria are better understood. Fungi synthesize enzymes such as cellulases, hemicellulases, and xylanases that are important for the breakdown of plant materials. Bacteria contribute to fatty acid metabolism, vitamin synthesis, short‐chain fatty acid production, microbial protein production, and a wide array of processes transforming the animal's diet to energy and nutrients. Within bacteria, the members of the Bacteroidota phylum are typically responsible for acetate and propionate production, while butyrate is typically produced by members of the Firmicutes phylum (Den Besten et al. 2013).

Many studies have reported correlations between the abundance of certain microbial populations and meat qualities. In a study comparing meat qualities and gut microbial communities from fecal samples of two cattle breeds (Angus and Xianjand brown cattle) raised on identical diets and conditions, Chen et al. (2022) found that Angus cattle, which had higher carcass weight and percentage of intramuscular fat, had significantly higher proportions of Bacteroidota (Chen et al. 2022). Within the Bacteroidota phylum, Prevotella copri was observed to also be significantly more abundant in Angus cattle. Ruminococcus gnavus and Blautia wexlerae were also observed to be significantly different between the cattle groups, both being more abundant in Angus cattle than the Xianjang brown cattle (Chen et al. 2022). Similarly, in their study relating rumen community populations with carcass qualities in cattle (*N* = 201), Holman et al. (2024) found a positive correlation between Prevotella and intramuscular fat (Holman et al. 2024). Further, positive correlations between marbling and Selenomonas have been reported. Selemononas has also been negatively correlated with methane emissions (Holman et al., 2024). Wang et al. (2020) investigated differences in ruminal populations and metabolic pathways in Holstein cattle fed different proportions of feed concentrate and forage. A high‐concentrate diet was found to increase the production of propionate and butyrate. The authors attributed the increase in propionate to higher metabolic activity in the succinate pathway, while the increase in butyrate was attributed to the transformation of butyryl CoA via acetate CoA transferase (Wang et al. 2020). Specifically, the genes related to enzymes such as pyruvate carboxylase, succinate‐CoA synthetase, and acetate CoA transferase were in higher abundance in samples from the high concentrate group (Wang et al. 2020). At the genus level, Ruminobacter, Selenomonas, Ruminobacter, Prevotella, and Vibrio were considered to be the main producers of propionate in the high concentrate group, with Prevotella also being the main producer of butyrate along with Bacteroides (Wang et al. 2020). Branched‐chain fatty acids, such as 4‐methyloctanoic acid, 4‐ethyloctanoic acid, and 4‐methylnonanoic acid, contribute to the distinct flavor of sheep and goat meat and milk (Watkins et al. 2021). The abundance of these compounds is related to enzymatic transformations of SCFAs, such as propionate, in the tissues. Though it is difficult to disentangle rumen populations from diet from animal given their interconnectedness, it is clear that ruminal communities do play a significant role in meat quality.

## 3 Multi‐Organ System Concept

### 3.1 Multi‐Organ Systems

Organ‐on‐a‐chip systems and conditioned media have both been used to simulate interactions between organs in vitro. Organ‐on‐a‐chip technologies have been utilized to study how tissues respond to different substances and conditions. Multi‐organ‐on‐a‐chip systems have further explored the dynamic interplay of physiological systems and offer promising platforms to replicate and better understand these synergies (Messelmani et al. 2023; Polidoro et al. 2021; Ronaldson‐Bouchard et al. 2022). These chip systems are more relevant to biomedical research than cultivated meat production, however, as they are designed for small‐scale, mechanistic work. In medical research, these chips are research tools wholly distinct from manufacturing and are inherently limited in scale. While media conditioning has been used in biomedical research and in the pharmaceutical industry, there is comparatively limited research on using media conditioning for cultivated meat. Combining these co‐culture or conditioned media systems with ruminal fermentation has never been evaluated. The proposed multi‐organ system (Rumen‐Liver‐Fibroblasts‐Target Cells) may address some of the challenges in the cultivated meat industry (Morikura et al. 2024; Ronaldson‐Bouchard et al. 2022; Shroff et al. 2023). Potential benefits of each component in supporting the culture of muscle and fatty tissues are outlined in Table 1.

**TABLE 1:** Support functions of multi‐organ system for cultivated meat production.

Support component	Key functions for media conditioning	Benefits to cultivated meat cultures
Fibroblasts	Secrete ECM proteins (collagen 1/3, fibronectin) and adhesion factors (Thummarati and Kino‐Oka 2020) Release paracrine growth factors (HGF, VEGF, bFGF, etc.) and cytokines (David et al. 2023; Ronaldson‐Bouchard et al. 2022) Can differentiate into adipocytes or modulate adipogenesis via signaling (Ma et al. 2024; Molina et al. 2021)	Provides structural matrix for cell attachment (replacing exogenous coatings) (David et al. 2023) Enhances myoblast differentiation and maturation via trophic signal (Hicks et al. 2012; Thummarati and Kino‐Oka 2020) Balances ECM deposition to avoid fibrosis; supports angiogenesis by producing VEGF (Thummarati and Kino‐Oka 2020)
Liver cells	Detoxification: Cytochrome P450 enzymes metabolize toxins; convert ammonia to urea (Getahun et al. 2019; Polidoro et al. 2021) Nutrient conversion: Gluconeogenesis (i.e., propionate to glucose); lipid processing (e.g., cholesterol, lipoproteins) (Nguyen et al. 2008) Secretion: IGF‐1 and IGF‐2, binding proteins, Produce low levels of albumin and other serum components (Morikura et al. 2024; Yamanaka et al. 2023)	Removes inhibitory metabolites like lactate and ammonia, and maintains cellular homeostasis (Getahun et al. 2019; Polidoro et al. 2021) Converts precursors to bioactive nutrients (e.g., SCFAs to glucose) (Nguyen et al. 2008) Supplies growth factors and carrier proteins (analogous to serum function)
Rumen microbiota	Fermentation: Digest complex carbs to SCFAs (acetate, propionate, butyrate); produce proteins and ammonia from dietary proteins or urea (Annison and Bryden 1998; Langda et al. 2020; Zhang et al. 2022) Vitamin synthesis: B vitamins (B2, B3, B6, B9, and B12) synthesized by microbes (Schnellbaecher et al. 2019; Seck et al. 2017) Flavor precursor generation: Produce branched chain fatty acids, odd chain fatty acids, and other metabolites from diet that can influence meat flavor (Annison and Bryden 1998; Franco‐Lopez et al. 2020; Watkins et al. 2021; Zhang et al. 2022)	Provides energy metabolites (SCFAs) that cells can use; butyrate acts as differentiation promoter at low levels (Holman et al. 2024; Wang et al. 2025) Natural vitamin provisioning reduces the need for added vitamin mix (cyanocobalamin, pantothenic acid, etc.) Generates compounds contributing to the aroma profile of meat (may allow diet related flavor tuning in vitro); fatty acid intermediates may improve nutritional profile (Annison and Bryden 1998; Franco‐Lopez et al. 2020; Watkins et al. 2021; Zhang et al. 2022)

By considering the multi‐organ system as a series of distinct modules rather than a literal multitude of cell types on a single device, one can explore how synergistic interactions of organs and cell types can be applied to cell culture with greater applicability to scaled manufacturing processes. These multi‐organ modules aim to simulate the biochemical environment that supports tissue growth in vivo by integrating key functions, such as microbial fermentation, nutrient conversion, and trophic support, into one overall system. For instance, gut‐ and liver‐inspired modules can work in tandem to process nutrients and eliminate metabolic waste, while stromal components can supply essential growth cues. As culture media are transferred among these modules, they could become progressively conditioned, resembling interstitial fluid and enhancing cell viability and differentiation. This multi‐organ approach, supported by multi‐directional media exchange and potential real‐time feedback, represents a step toward mimicking the complexity of an animal's internal systems to improve the quality and functionality of cultivated muscle tissue (Andria et al. 2010; Chu et al. 2024; Shen et al. 2019; Van et al. 2024).

### 3.2 Conceptual Applications of Conditioned Media and Multi‐Bioreactor Systems

**FIGURE 2:**
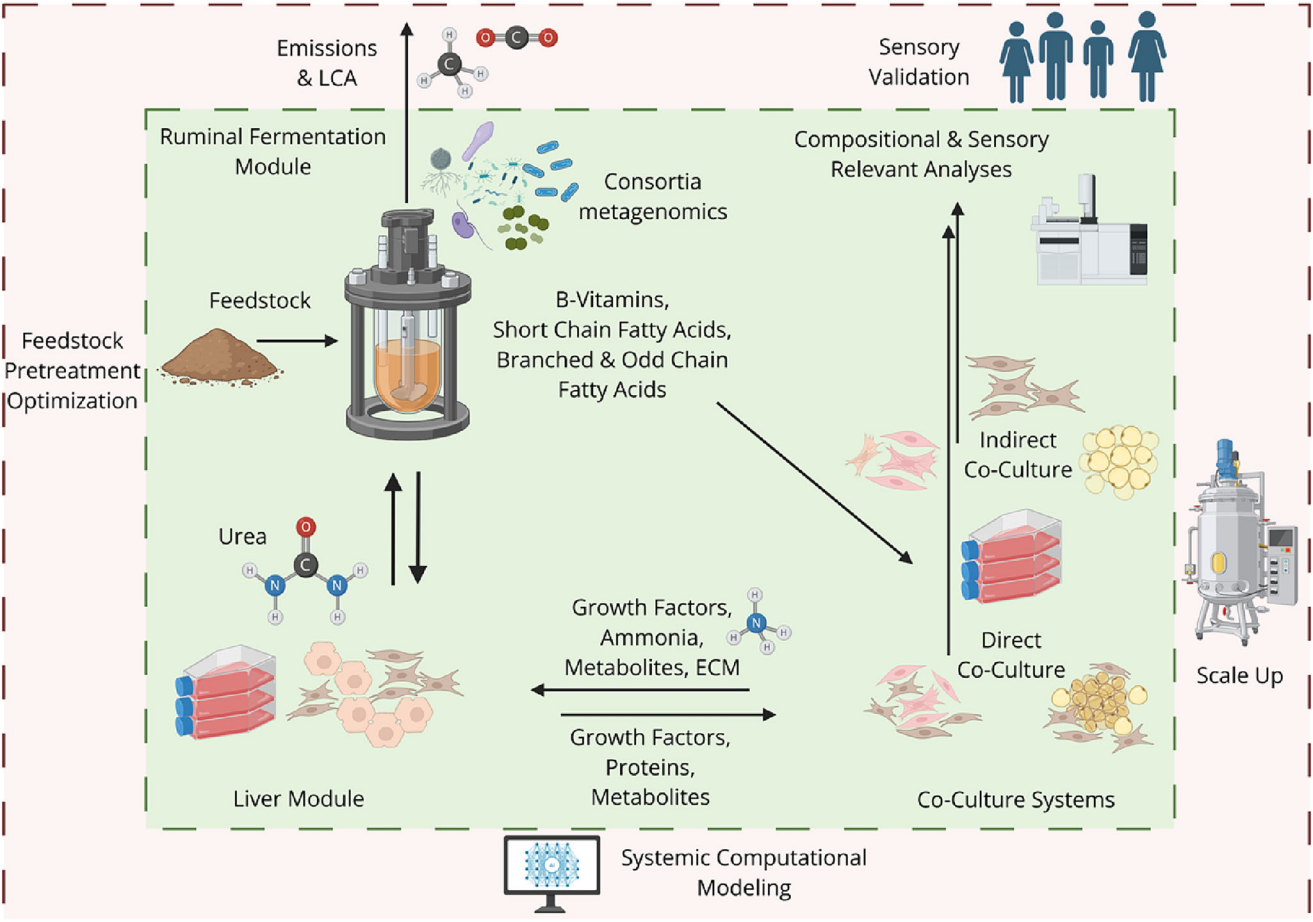
Example of interconnected bioreactor series for cultivated meat production. Items within the green box (interior) are considered to be key processes, metabolites, and analyses to the proposed system. The red box (exterior) are important areas for future research and development.

A visual representation of a potential interconnected system is shown in Figure 2. The proposed system is conceptual at this stage, with many challenges envisioned in its implementation. Some challenges will need to be worked out empirically based on the specifics of the application (e.g., animal species and tissue type being produced), though general strategies can be taken to enable researchers to engage in this work. With a large number of potential cell types and organisms involved, there must be systems in place to avoid cross‐contamination of living organisms or deleterious substances across the modules.

The ruminal fermentation module should be physically separate from the other elements to prevent contamination of other cultures and because direct cell‐cell signaling or contact is not believed to be a potential benefit. Ruminal fermentation could be operated in an anaerobic bioreactor system, wherein a rumen‐derived microbial consortia could break down a feedstock, culture media, waste from other systems, or a combination thereof. Ruminal post‐biotics can be separated from the microbial biomass via downstream processes like centrifugation and sterile filtration to remove any living organisms or spores before being concentrated or utilized as a conditioned medium for mammalian cell cultures. It may be necessary to further ready the postbiotics with ultrafiltration to reduce the risk posed by endotoxins and other substances that may negatively impact the cellular modules or the eventual human consumers. Successful development of such a system could enable a degree of control over final product flavor, akin to the impact on flavor profiles of meat from animals on different diets (e.g., grain‐fed, grass‐fed).

A liver cell module wherein hepatocytes or other liver cells are directly co‐cultured with fibroblasts to provide structure and improve and prolong hepatic function could be effective at generating a conditioned medium rich in growth factors and fatty acids, as well as cholesterol and albumin to some degree (Bale et al. 2015; Miranda et al. 2010; Polidoro et al. 2021; Yamanaka et al. 2023). Whether the main biomass tissue type is muscle tissue or adipose tissue, both could similarly benefit from either direct or indirect co‐culture with fibroblasts. Both example biomass types would also generate ammonia as a waste product. By connecting the liver module with the tissue biomass module, they could complement each other by the exchange of growth factors and nutrients, while the liver module could detoxify ammonia by converting it to urea, which could itself be returned to the rumen module as an additional nitrogen source. Ultrafiltration should not be necessary for transfer of conditioned media between liver modules and main cell modules, though sterile filtration will still be critical to maintaining healthy, independent cellular processes. Keeping the modules physically separated would allow for potentially easier or more robust sterilization procedures before media is transferred, but physically separate systems would also negate the theoretical benefit of waste product removal without the need for media changes as discussed previously. Thus, early‐stage work in the development of the multi‐organ system would benefit from fully separated modules, while later‐stage work could attempt to take advantage of waste removal in a more well‐defined, interconnected bioreactor series.

### 3.3 Anticipated Challenges of Multi‐Organ Bioreactor Series Systems

While the vision of a multi‐organ bioreactor series production system is compelling, it will require substantial development and optimization to meet the scale and cost needs of food manufacturing. Some of the key hurdles and future research directions include standardization of co‐culture platforms, scaling co‐culture systems, safety and regulatory considerations, and integration of intelligent control systems, which are discussed here.

#### 3.3.1 Custom and Variable Design for Each Module

Each cell and tissue type has its own distinct requirements (e.g., media composition, oxygen levels, optimal culture duration) and limitations (e.g., stress tolerances, lifespans, and phenotypic stability) that must be considered when designing the system. Furthermore, new immortal cell lines will almost certainly need to be developed for each species of interest given the lack of suitable, commercially available lines. For example, immortalized liver epithelial cell lines such as HepG2 and RL34 have been successfully used to condition media for mammalian cultures, but they are derived from human and rat tissues, respectively (Morikura et al. 2024; Yamanaka et al. 2023). For food production purposes, it will be ideal to use a cell line from the same species as the main cellular tissue being produced rather than a separate species. With regard to bioprocess, even in a simplistic three‐bioreactor version of this multi‐organ production system, the same bioprocess would not be applied to all three bioreactors. Additionally, there is inherent variability in biological systems even under controlled conditions. Creation of a semi‐closed loop system among the various modules will be complex. Though the interplay of the modules is desired, they must be distinct enough to allow for some degree of corrections, control, and processing as the inputs and outputs are moved around. Considerations for designing complex bioreactor systems have been detailed out by Mandenius (2018), though not for cultivated meat or food production purposes (Mandenius 2018).

#### 3.3.2 Differential Growth Rates of Cell Types

The major role of the liver cells and fibroblasts in the co‐culture system described is providing key signals and factors to support the attachment, expansion, proliferation, and differentiation of the target cells (e.g., adipose or muscle). However, this co‐culture must be maintained at the desired ratios to the target cells, not allowing them to substantially slow down or die off as well as not allowing them to overtake the target cells by growing too rapidly. The synergies of the multi‐organ system are intended to help with inadequate growth by promoting growth and phenotype stability. To avoid one cell type overtaking another, growth can be inhibited through ionizing technologies (such as gamma irradiation) and chemical treatments (such as mitomycin‐C) (Llames et al. 2015). For food applications, the ionizing technology would likely be preferred so as to not introduce a new chemical hazard to the product. Furthermore, feeder layer cells are typically treated to the point that their growth is negligible, which may be undesirable. It is possible that either a less intense initial treatment or inclusion of additional non‐treated cells could help achieve the desired growth rates.

#### 3.3.3 Scale Appropriate Support Systems

The multi‐organ system becomes less feasible if the resources required to operate it are too great. Ultimately, the liver module and the rumen module are support systems to produce the cultivated tissues and fat. As such, they should not need to be operated at the same scale as the tissue/fat system. While it is ideal that all systems be as productive per unit volume or material input as possible, it is also important that these support systems be efficient enough that they can fulfill their respective roles in substantially smaller scale systems than the main production system.

#### 3.3.4 Cost‐Effective Processes

The practicality of the support systems is also dependent on the level of downstream processing required. The benefit of the bio‐inspired systems is both in the generation of diverse metabolic products and in the lack of need for intense purification processes. With regard to the production of SCFA, Rodriguez et al. (2014) describe that the cost associated with biological production of propionate is largely driven by the use of purified sugars and the extent of downstream processing. Use of less refined corn mash as a substrate was modeled to be 30‐fold cheaper than processes using pure substrate (Rodriguez et al. 2014). Furthermore, many of the microbes known to be good producers of SCFA such as propionic acid are rumen bacteria (Gonzalez‐Garcia et al. 2017). In their work conditioning media for mammalian cultures, Yamanaka et al. (2023) utilized relatively simple mineral medium with algal biomass extract for their culture of the liver cells (Yamanaka et al. 2023). As discussed previously, use of less refined raw materials is expected to substantially reduce costs associated with production. The potential use of other low‐cost agricultural products or agricultural waste stocks such as corn stover could be assessed, though algae remains of interest. The cellulolytic capabilities of the rumen module may be able to derive at least some of an agriculture waste media, or the pretreatment of a feedstock may be able to generate amino acid‐ and glucose‐rich media for use in this system. While we have not modeled the multi‐organ system at this early stage, the potential to use inexpensive agricultural streams and minimal downstream purification give some credence to the concept.

#### 3.3.5 Non‐Standardized Ruminal Communities

Rumen microbial consortia are highly variable, diverse, dependent on many factors (e.g., species, feed composition, time since feeding, and environmental conditions), and are notoriously difficult to culture all of (or even the majority of) the microbial species thought to be present in vivo (Botero Rute et al. 2024; Deitmers et al. 2022; Holman et al. 2024; Morgavi et al. 2013). Efforts to standardize procedures and testing methodologies have been proposed, including the use of the rumen simulation technique (RUSITEC) (Deitmers et al. 2022; Deitmers et al. 2024). However, even with standardization, challenges associated with culturing many of the microbial types persist, and the results of in vitro consortia fermentation are different from in vivo (Botero Rute et al. 2024). As environmental factors and metabolite interactions occur within such diverse communities, variability in metabolism or consortia populations likely compounds variability in each other.

#### 3.3.6 Feedstock Selection and Treatment

The nutrients provided to the rumen consortia affect the abundance of different populations, whether those nutrients are in the form of feed or culture media (Annison and Bryden 1998; Botero Rute et al. 2024; Zhang et al. 2022). Additionally, if feed or feedstocks are utilized for fermentation, how the feedstock is prepared or pretreated will also have an effect, as there can be alterations to physical and chemical structures that are relevant to microbial and enzymatic breakdown processes. Pretreated feedstocks can be more readily accessed by enzymes or microbes when they are partially physically broken down or chemically modified, potentially speeding up the desired fermentation process (Fan et al. 2025). With the intended use ultimately being food for human consumption, it would be important to select feedstock pretreatment methods that do not introduce new chemical hazards to the material (Singh et al. 2016). Physical pretreatments or technologies such as electron beam (eBeam) may be most ideal for these purposes. Additionally, while minimizing variability is generally desirable, the varied outcomes of feeding animals different diets do produce variable qualities in meat and dairy products that are positive and contribute to the breadth of high‐quality foods available to consumers with different preferences (Chail et al. 2016; Dinh et al. 2021). Use of varied feedstocks may allow for cultivated products to continue to provide the breadth of high‐quality and nuanced products available in conventionally produced meats (Chail et al. 2016; Dinh et al. 2021).

#### 3.3.7 Food Safety and Regulatory

At this time, regulations do not exist for use of the proposed multi‐organ system for food production. However, regulatory frameworks do exist globally for both the use of cell culture for the production of food and the use of microbial fermentations to produce ingredients and culture media components. Ruminal fermentation components will be the most novel aspect from a regulatory view. Looking to current regulations for insight, it is expected that good documentation of the sources of the rumen microbes, demonstration of stability and control over the process, thorough characterization of the end product, and specific testing for pathogens, toxins, contaminants, and other agents known to be potential hazards to humans will be priorities to regulators. Construction of a rumen microbial community using strains that are shown to be nonpathogenic, or even which are already found in other food products, could make the regulatory application process smoother than if a community directly isolated from animal rumens were to be used.

#### 3.3.8 Greenhouse Gas Emissions

In addition to the SCFAs and B‐vitamins described above, ruminal fermentation produces greenhouse gases (GHGs), notably methane and CO_2_ (Holman et al. 2024). In fact, ruminant livestock production is one of the major sources of greenhouse gas emissions (FAO 2023; Tuomisto and Ryynänen 2024; Xu and Lan 2016). Given that one of the primary goals of developing cultivated meat is to mitigate the environmental impact of meat production, it would be ideal to understand the extent of production of GHGs from a proposed ruminal fermentation module in a cultivated meat production system by conducting a life cycle assessment (LCA). Recent research has suggested that GHG emissions from ruminants could be reduced by altering the diet of the animal. Specifically, incorporation of a red microalgae (*Asparagopsis taxiformis*) was found to dramatically reduce methane production in Angus‐Hereford cattle (Roque et al. 2021). If unacceptably high levels of methane are observed to be produced in a rumen fermentation module, incorporation of red microalgae could be investigated to see if it has similar effects. Alternatively, gas emissions from the module could theoretically be captured to be utilized for other valorization processes.

#### 3.3.9 Modeling Complexity Across Systems

To be able to meaningfully benefit from the complex set of systems discussed, one will need to have a robust understanding of how each module works individually and in concert with the other modules. Such an understanding will need to be built from robust datasets from many experiments. The sheer breadth of variables to track and the complex interactions of these biological systems necessitate computational modeling tools to take full advantage of the data generated. Beyond computational modeling, AI and machine learning approaches are being increasingly applied to predict optimal operating conditions, media formulations, or metabolic responses. These approaches allow researchers to refine biological and engineering parameters, making the system more predictable, scalable, and aligned with the complex demands of cultivated meat production. Given the early, conceptual nature of the proposed multi‐organ system, modeling of system interactions or mass‐balance calculations has not yet been attempted.

## 4 Conclusion

Ruminal fermentation as part of a multi‐organ approach to cultivated meat production is a compelling concept. Product quality is one of the main drivers of consumer purchases, and yet very little cellular agriculture research has been published in this area. As such, development of a greater understanding of cultivated meat quality is of great importance. Though the industry this proposed system seeks to support is currently broadly focused on reducing costs and dramatically scaling up, the complexity added by the multi‐organ concept need not work counter to the commercial practicalities of production. Use of a bioreactor series, keeping modules physically distinct, allows for greater control and refinement of unit operations. The interplay of various cell types, metabolites, and cellular products is well established. A bio‐inspired multi‐organ cultivation systems approach may allow for reduced or obviated need for added media components and improved product qualities such as flavor, texture, and nutrition.

To operationalize the multi‐organ bioreactor series concept, several priority areas require interdisciplinary research and investment, including microbial community development, cell line development, bioprocess development, extensive metabolomics and compositional analyses, computational modeling, pilot‐scale prototyping, and sensory and nutritional validation. Realization of a multi‐organ system for cultivated meat will require the convergence of scientific disciplines as well as collaboration across academia, startups, and public‐sector agencies. With support from global research networks, industry stakeholders, and governmental bodies, this systems‐level approach may further the transition from concept to a transformative platform for delicious, nutritious, sustainable protein production.

## Author Contributions

**Morgan Rease**: conceptualization, investigation, writing – original draft, methodology, validation, visualization, writing – review and editing, formal analysis, and data curation. **Wasitha P.D.W. Thilakarathna**: writing – original draft, methodology, validation, visualization, writing – review and editing. **Chandni Praveen**: writing – original draft, methodology, validation, visualization, writing – review and editing. **Suresh Pillai**: conceptualization, investigation, funding acquisition, writing – original draft, methodology, validation, visualization, writing – review and editing, supervision, and resources. **Reza Ovissipour**: conceptualization, investigation, funding acquisition, writing – original draft, methodology, validation, visualization, writing – review and editing, formal analysis, project administration, supervision, and resources.

## Conflicts of Interest

Morgan Rease reports on a relationship with UPSIDE Foods Inc. that includes equity or stock ownership. UPSIDE Foods is a cultivated meat company.
